# Serum procalcitonin improves diagnosis of infectious complications after CRS/HIPEC

**DOI:** 10.1186/s12957-022-02884-9

**Published:** 2023-01-12

**Authors:** Lilian Roth, Dilmurodjon Eshmuminov, Linda Russo, Felix Laminger, Friedrich Kober, Sebastian Roka, Kuno Lehmann

**Affiliations:** 1 Surgical Oncology Research Laboratory, Department of Surgery & Transplantation, University Hospital of Zurich, Raemistrasse 100, CH-8091 Zurich, Switzerland; 2 Department of Surgery, Center for Peritoneal Carcinomatosis, Hanusch-Krankenhaus, Vienna, Austria

## Abstract

**Background:**

Cytoreductive surgery (CRS) and hyperthermic intraperitoneal chemotherapy (HIPEC) improve the survival of selected patients with peritoneal metastasis. A major cause of treatment-related morbidity after CRS/HIPEC is infection and sepsis. HIPEC alters the diagnostic sensitivity and specificity of blood and serum markers and therefore has an impact on early diagnosis of postoperative complications. This study aimed to assess the sensitivity and specificity of blood and serum markers after CRS/HIPEC.

**Methods:**

Patients from two centers, operated between 2009 and 2017, were enrolled in this study. Perioperative blood samples were analyzed for white blood cells (WBC), C-reactive protein (CRP), and procalcitonin (PCT); postoperative complications were graded according to Clavien-Dindo and infectious complications according to CDC criteria.

**Results:**

Overall, *n*=248 patients were included with peritoneal metastasis from different primary tumors treated by CRS/HIPEC. Depending on the applied HIPEC protocol, patients presented a suppressed WBC response to infection. In addition, a secondary and unspecific CRP elevation in absence of an underlining infection, and pronounced after prolonged perfusion for more than 60 min. PCT was identified as a highly specific — although less sensitive — marker to diagnose infectious complications after CRS/HIPEC.

**Discussion/conclusion:**

Sensitivity and specificity of WBC counts and CRP values to diagnose postoperative infection are limited in the context of HIPEC. PCT is helpful to specify suspected infection. Overall, diagnosis of postoperative complications remains a clinical diagnosis, requiring surgical expertise and experience.

**Supplementary Information:**

The online version contains supplementary material available at 10.1186/s12957-022-02884-9.

## Synopsis

HIPEC treatment after CRS influences the accuracy of common inflammatory parameters to diagnose a postoperative infectious complication. The additional determination of procalcitonin increases the specificity in the diagnosis.

## Introduction

Cytoreductive surgery (CRS) and hyperthermic intraperitoneal chemotherapy (HIPEC) have become an accepted component of multimodal therapy of peritoneal metastasis. While CRS refers to a systematic and radical resection of visible peritoneal implants, HIPEC is an innovative strategy to control microscopic disease by an intraoperative, heated chemo-perfusion. Over the last years, the concept of CRS/HIPEC changed the landscape of treatment for peritoneal metastasis and demonstrated impressive survival rates, e.g., for colorectal [1], gastric [2], or ovarian [3] metastasis. Despite all advances made in the treatment of peritoneal metastasis, HIPEC is still performed in various ways, and several parameters remain poorly defined, varying among centers. For example, this includes the treatment duration, the degree of hyperthermia, or the substances or combinations used, which are likely to change in the future. Today, many HIPEC protocols use combinations of mitomycin C/doxorubicin, oxaliplatin or cisplatin, and use a temperature range between 41 and 43°C for 30 to 90 min.

CRS/HIPEC, a radical and potentially curative treatment modality, is associated with the risk of treatment-related morbidity and mortality. By far the major contribution relates to the surgical procedure. However, HIPEC may add to the overall morbidity, and have some specific morbidity. For example, mitomycin C is known to have a negative impact on WBC counts in up to 39% of patients [4, 5], oxaliplatin may be associated with hemorrhagic complications [6], and cisplatin can induce severe nephrotoxicity [6, 7]. A recent study from the USA compared treatment-associated morbidity of CRS/HIPEC with other major surgery, e.g., liver resection, Whipple’s procedure or esophagectomy, and identified an overall lower morbidity and a low mortality rate of 1.1% [8]. After CRS/HIPEC, the major cause for treatment-related death is sepsis and infection [9]. Early recognition of complications has been recently defined as a major factor to reduce failure-to-rescue after CRS/HIPEC [10]. Therefore, a reliable diagnosis of infectious complications after CRS/HIPEC is crucial. Although the clinical picture of patients remains the fundament of surgical diagnosis of postoperative complications, blood parameters may be helpful to screen or specify.

We reported in a previous report, that HIPEC can provoke a systemic inflammatory response [11]. This is very likely to have an impact on sensitivity and specificity of laboratory values, e.g., WBC counts, C-reactive protein, or procalcitonin. In the present study, we assessed the role of standard blood parameters (WBC counts, C-reactive protein, procalcitonin) to diagnose postoperative infectious complications after CRS/HIPEC.

## Material and methods

### Patients and ethics

The study includes patients from two centers (University Hospital Zurich, Switzerland, and Hanusch Krankenhaus, Vienna, Austria) operated between 2009 and 2017. The study protocol was approved by the ethical committee (KEK-ZH-Nr.2017-01656) and registered at clinicaltrials.gov (NCT02741167).

### Surgery and perioperative management

All patients were discussed prior any treatment in a multi-disciplinary tumor board. Extra-abdominal tumor was excluded by ^18^FDG-PET/CT or contrast-enhanced thoraco-abdominal CT. Patients received standard of care pre- and postoperative chemotherapy according to their tumor entity and international guidelines. Anesthesia was conducted with propofol and volatile anesthetics combined with thoracic epidural anesthesia as described previously [12]. CRS was performed according to international standards, and defined as radical (CC-score 0) if no macroscopic residual tumor was visible, except for pseudomyxoma, where a CC-1 score (<0.25cm remnant macroscopic tumor) was accepted [13]. For appendix and colorectal tumors, peritoneal dialysis solution with mitomycinC (30mg/m^2^ body surface area, BSA according to the Mosteller formula) in combination with doxorubicin (15mg/m^2^ BSA) was applied at 42°C for 90 min, or oxaliplatin (300-400mg/m^2^ BSA) as a single agent at 43°C for 30 min. Patients with mesothelioma or ovarian cancer were treated with a cisplatin-based regimen (75mg/m^2^ BSA) at 42°C for 90 min. In 2016, the type of protocol used for the appendix and colon cancer changed in both centers from mitomycinC/doxorubicin to oxaliplatin, which was then consistently used for these tumors until the end of the study. Patients received pre/intraoperative antibiotic prophylaxis (cefuroxime 1.5g, metronidazole 500mg) which was not continued to the postoperative phase.

### Serum probes

C-reactive protein (CRP), white blood cell (WBC) counts, and procalcitonin (PCT) were measured in blood samples by the clinical laboratory service on a daily routine basis prior to open surgery or CRS/HIPEC and for the 14 consecutive postoperative days or until the date of discharge. A positive event for WBC counts or CRP and PCT levels was defined if the value at day 8 was higher or equal compared to the value at day 5. In addition, only WBC counts above the normal range (>10G/l) were considered as a positive event.

### Definition and diagnosis of postoperative infection

For the grading of complications, the Clavien-Dindo classification was used [14]. Definition of infectious complications was done according to the Center for Disease Control and Prevention (CDC) definitions [15]. Patients after CRS/HIPEC were visited and examined daily. In case of clinical symptoms or signs of infection, urine and central catheter tips were sent for cultures. Imaging studies, usually an abdominal CT, were performed if CRP levels increased >30% after postoperative day 4.

### Statistical analysis

Continuous variables were compared with the Student *t*-test, the Mann–Whitney *U*, or the Wilcoxon test, where appropriate. Fischer’s exact test was used to compare differences among proportions derived from categorical data. Normally distributed data are shown as mean +/-SD, non-normal variables as the median and interquartile range (IQR). Missing values in the dataset were excluded. All *p* values were two-sided and considered statistically significant if p≤0.05. Statistical analysis was performed using SPSS version 25 and GraphPad Prism version 8.0. Sensitivity and specificity of each diagnostic parameter were determined by the kinetics between postoperative day 5 and day 8 and the number of patients with an infectious versus non-infectious complication.

## Results

Overall, *n*= 248 patients after CRS/HIPEC were included in this analysis. Overall, 41% (*n*=145) of patients had any complication, in 10% (*n*=25) of patients major morbidity (≥Clavien Grade 3b) was observed, and one patient died (Table 1). For HIPEC, three protocols (mitomycin C, oxaliplatin and cisplatin) were used. Patients differed in terms of primary tumors and median operation time, but not the PCI (Table 2). With the primary goal to test the diagnostic accuracy of serum parameters, we first assessed if WBC counts and serum CRP are able to diagnose postoperative infectious complications.

**Table 1:** Patient characteristics

	CRS/HIPEC
Number of patients	248
Age	54 (46–63)
Gender (male/female)	141(57%)/107(43%)
Preoperative systemic chemotherapy	106 (43%)
Anastomosis (number)	1 (0–2)
Complications (Clavien Dindo)	
none	145 (59%)
Grade I	11 (4%)
Grade II	42 (17%)
Grade IIIa	22 (9%)
Grade IIIb	21 (8%)
Grade IVa	4 (2%)
Grade IVb	0
Grade V	3 (1%)
Infectious complications	61 (25%)
Superficial	16/61 (26%)
Deep	2/61 (3%)
Organ space	43**/**61 (71%)

Patient characteristics for patients after CRS/HIPEC. Categorial data are presented as absolute numbers with percentage and nominal data as median and IQR

**Table 2:** Comparison of patient characteristics between HIPEC protocols

	Mitomycin C	Oxaliplatin	Cisplatin	*p*-value
Number of patients	123	48	77	
HIPEC				
Perfusion time (min)	90	30	90	
Temperature (°C)	42	43	42	
Primary tumor				0.000
Colorectal	44 (36%)	25 (52%)	7 (9%)	
High-grade appendix	32 (26%)	19 (40%)	2 (3%)	
Low-grade appendix	38 (31%)	0		
Mesothelioma	0	0	14 (18%)	
Others	9 (7%)	4 (8%)	54 (70%)	
PCI	10 (4–21)	8 (3–17)	9 (4–20)	0.17
Operation time (min)	540 (445–685)	361 (284–479)	405 (281–546)	0.000
Splenectomy	26 (21.2%)	9 (18.8%)	18 (23.4%)	0.50
ICU stay	1 (1–2)	1 (1–4)	2 (1–5)	0.000
Hospital stay	18 (13–25)	17 (15–31)	16 (12–20)	0.035
Infectious complications	39 (32%)	10 (21%)	12 (16%)	0.028
Superficial	7/39 (18%)	4/10 (40%)	1/12 (8%)	0.53
Deep incisional	1/39 (3%)	1/10 (10%)	1/12 (8%)	1.00
Organ/space	31/39 (79%)	5/10 (50%)	10/12 (84%)	0.000
Intestinal leak	7/31 (26%)	0		
Urinary infection	5/31 (16%)	0	1/10 (10%)	
Positive blood culture	8/31(26%)	1/5 (20%)	2 (20%)	
Pneumonia	3/31 (9%)	1/5 (20%)	4 (40%)	
Intraabdominal abscess	4/31 (13%)	2/5 (40%)	2 (20%)	
Infected pancreatic fistula	1/31 (2%)	1/5 (20%)	1 (10%)	
Bacterial peritonitis	2/31 (6%)	0	0	
Other ^a^	1/31 (2%)	0	0	

**Fig. 1:**
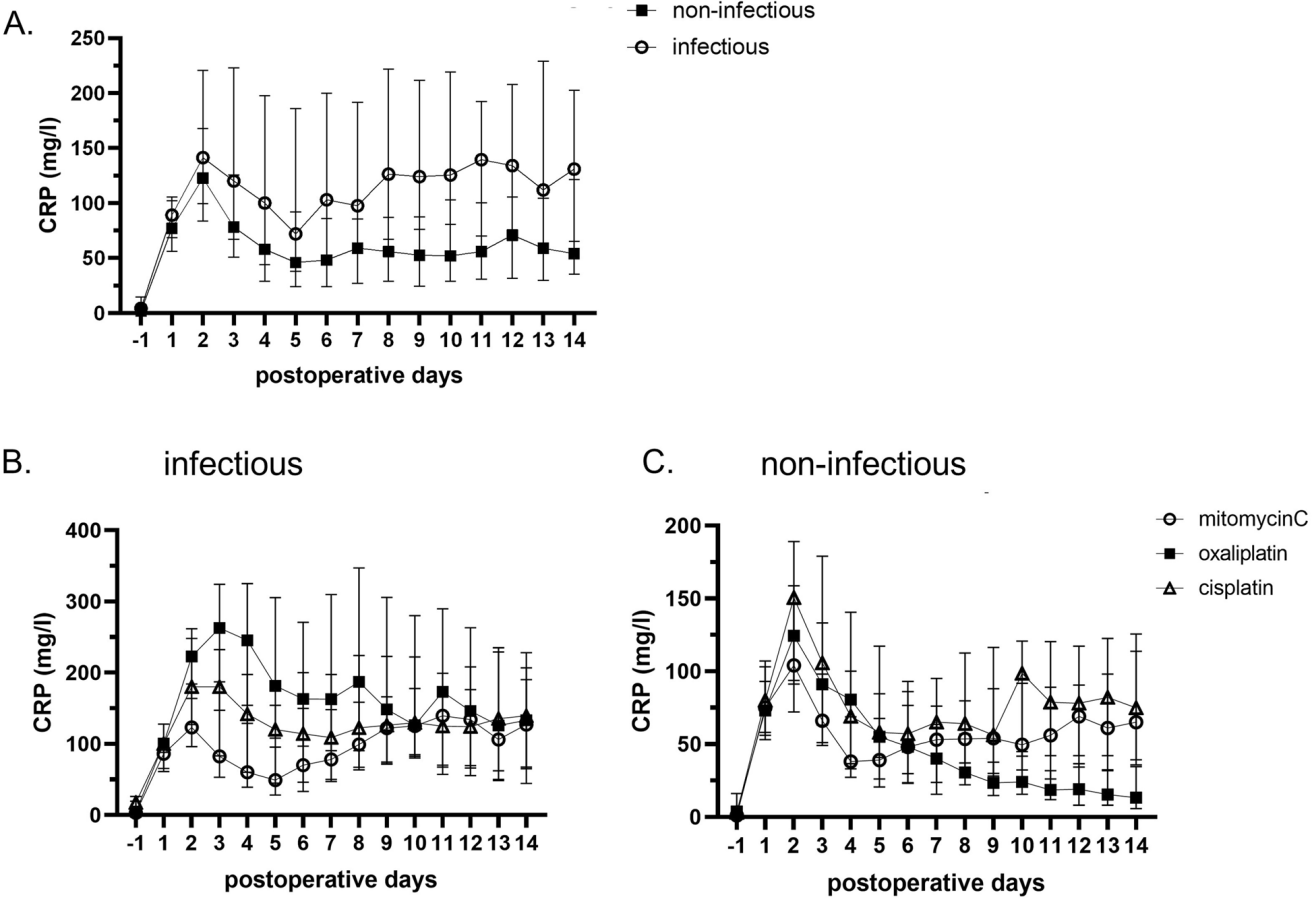
HIPEC treatment influences postoperative CRP levels. CRP levels after CRS/HIPEC do not return to normal, even without any infectious complication (**A**). With any infectious complication, CRP increases after HIPEC performed with any of the three protocols (**B**). In absence of infectious complications, CRP increases after mitomycinC and cisplatin based HIPEC (**C**), while returning to normal values after HIPEC performed with oxaliplatin (**C**). The graphs illustrates the postoperative CRP values, plotted as median and IQR

**Fig. 2:**
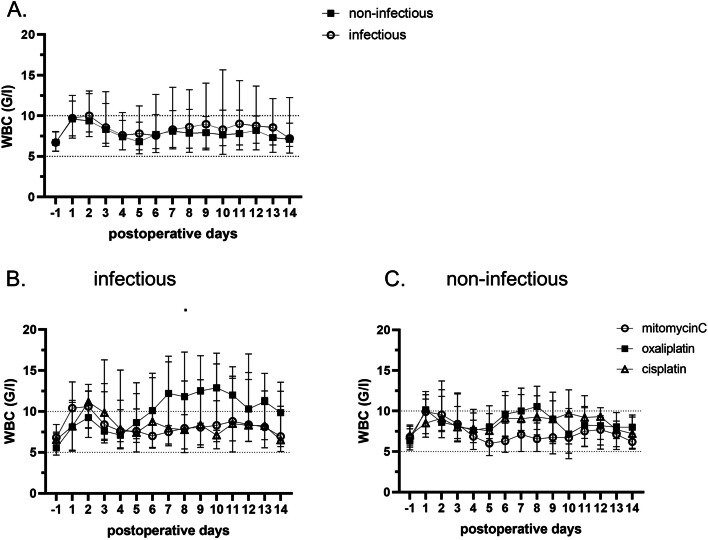
HIPEC treatment suppresses WBC counts. Postoperative WBC counts remain within normal ranges, even in presence of infectious complication (**A**). WBC`s remain reactive to infection only after oxaliplatin based HIPEC (**B**). Without any infectious complications, the WBC`s counts remain within the normal ranges of 5–10 G/l illustrated for each HIPEC protocol (**C**)

**Fig. 3:**
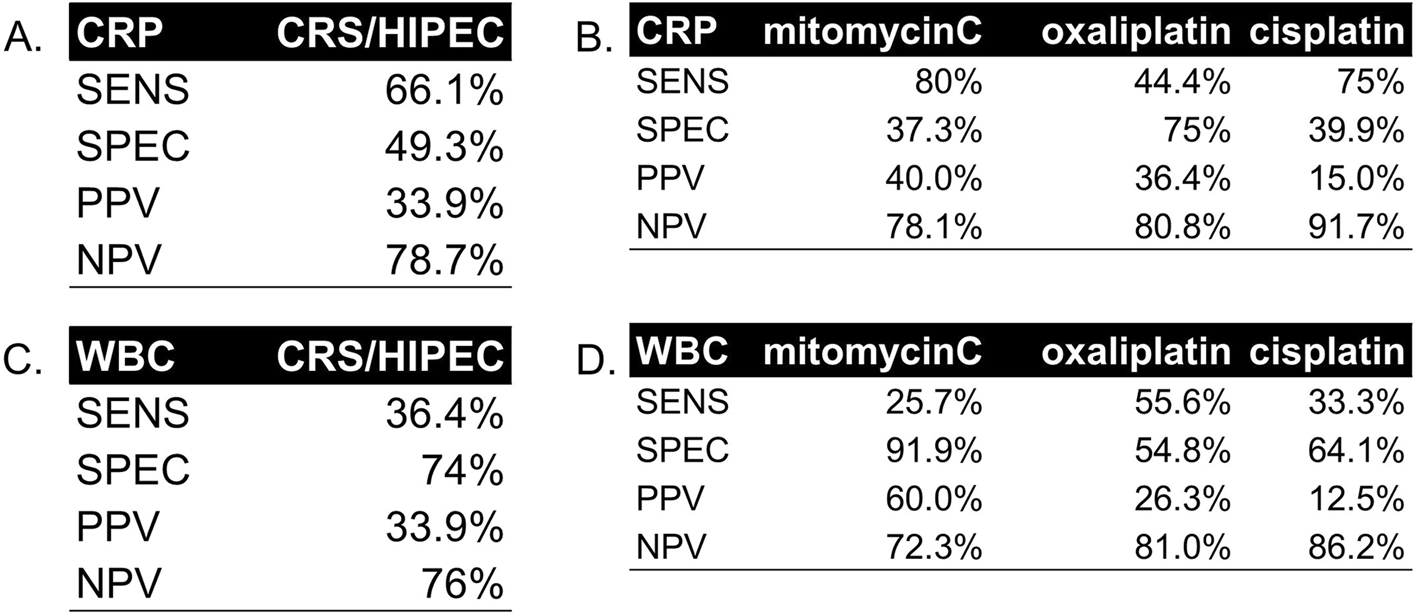
Sensitivity and Specificity of CRP and WBC counts after HIPEC. The specifitiy of CRP after CRS/HIPEC is only 49.9% (**A**). Specificity is reduced after mitomycinC and cisplatin based HIPEC to 37.3% and 39.9% respecitivly (**B**). WBC counts demonstrate a low sensitivity of 36.4% in general (**C**), pronounced after prolonged protocols with mitomycin C or cisplatin (**D**). SENS, sensitivity; SPEC, specificity; PPV, positive predictive value; NPV, negative predictive value

**Fig. 4:**
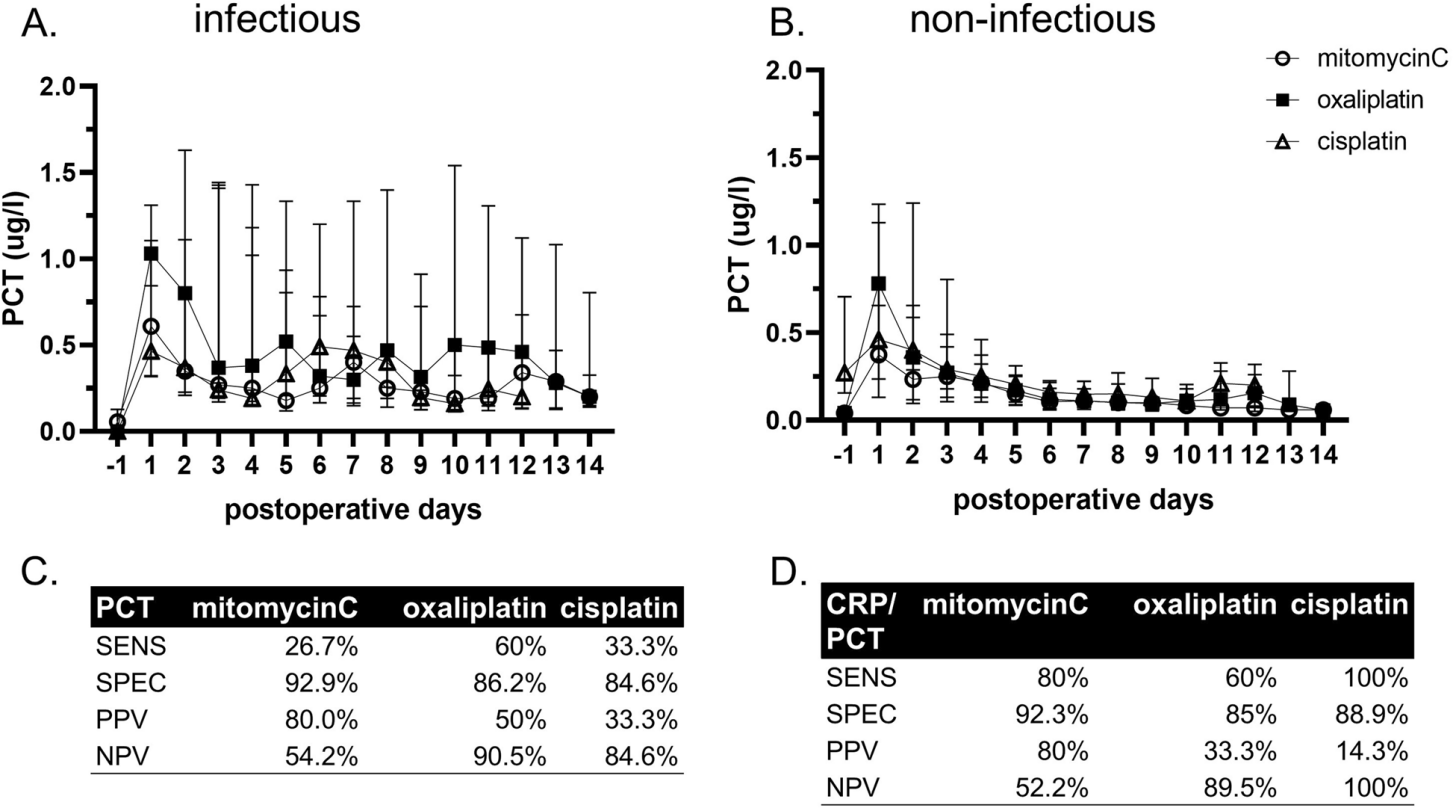
Serum procalcitonin (PCT) improves specificity to diagnose infectious complications. PCT is highly specific to detect postoperative infection after HIPEC, independent of the protocol. SENS, sensitivity, SPEC, specificity; PPV, positive predictive value; NPV, negative predictive value

Patients after CRS/HIPEC were assessed according the HIPEC protocol, which differ with regard to the drugs used for HIPEC and the perfusion time. Particularly the early kinetic of inflammatory markers (Figs. 1, 2, 3 and 4) should be read with the information that the groups differ the primary tumor and operation times. ^a^Other infectious complications include cholangitis and colpitis. Categorial data are presented as absolute numbers with percentage and nominal data as median and IQR

### Low specificity of CRP and low sensitivity of WBC counts after CRS/HIPEC

In general, CRS/HIPEC is associated with a low specificity of CRP to diagnose an infectious complication during the postoperative course. The reason for this is the secondary peak of CRP between days 5 and 8, also present in absence of any infection (Fig. 1A). Generally, the CRP levels after HIPEC remained elevated during the observation time of two weeks. In contrast to CRP, WBC counts remained within a normal range, even in presence of postoperative infections (Fig. 2A), which results in a very low sensitivity of 36.4% (Fig. 3C).

### CRP levels are unspecific after 90-min platin-based protocols

After the observation that HIPEC can elevate postoperative CRP levels in absence of infection and suppress WBC counts in response to infection, we next explored whether these effects depend on the HIPEC protocol. In this study, HIPEC was performed with oxaliplatin (*n*=48), mitomycinC/doxorubicin (*n*=123), or cisplatin (*n*=77). Upon infection, CRP levels increased after any protocol as expected (Fig. 1A). In contrast, in patients without infection, patients after a 90-min protocol with mitomycinC or cisplatin, the above-mentioned secondary CRP peak was observed between postoperative day 5 and day 8 (Fig. 1C). Consequently, CRP levels demonstrated a poor specificity (37–40%) to diagnose postoperative infection in these two protocols (Fig. 3B). As a consequence, infection was suspected and over-diagnosed in 16% (13/84) of patients after HIPEC with mitomycinC, who underwent an abdominal CT scan without a diagnosis of complications

### White blood cell counts to diagnose infection after HIPEC

In contrast, WBC counts have a moderate sensitivity to diagnose infection (Fig. 3C). This effect is more pronounced after HIPEC with mitomycinC or cisplatin (Fig. 2B), where WBC kinetics show no response to infection. This is different after oxaliplatin-based HIPEC, where WBC counts are able to react to infection, resulting in a higher sensitivity (Fig. 3D) of this marker. Overall, WBC counts seem to have only a moderate utility to diagnose infection after the CRS/HIPEC.

### Serum procalcitonin (PCT) improves specificity to diagnose infectious complications

Given the low specificity of CRP to diagnose postoperative infection after CRS/HIPEC, we assessed the diagnostic value of PCT in this setting. PCT values reacted similarly to infection, regardless of the perfusion protocol (Fig. 4A), and did not show a nonspecific reaction as seen for CRP (Fig. 4B). Despite a low sensitivity, PCT demonstrated a high specificity of >85% to diagnose infection for all protocols (Fig. 4C). Assessment of PCT in addition to CRP can be helpful to distinguish between infectious complications and a non-specific CRP increase, particularly for protocols with prolonged perfusion times (Fig. 4D).

## Discussion

This study highlights the specific role of HIPEC on the pathophysiology of postoperative serum inflammatory parameters. We observed that certain HIPEC protocols can suppress the WBC response to infection and may cause secondary and unspecific CRP elevations without underlining infection. This has a major impact on the sensitivity of WBC counts or the specificity of CRP values. We observed that this effect depends on the specific HIPEC protocol and seems more pronounced after prolonged perfusion for 60 min or more. To overcome this diagnostic limitation, we assessed the role of PCT, which was identified as a highly specific — although less sensitive — marker to diagnose infection. These findings may help to discriminate and diagnose infectious complications in the setting of CRS/HIPEC.

Due to the complexity of the procedure including HIPEC which induces additional tissue damage and inflammation, diagnosis of postoperative infection can be challenging. Knowledge about the potential suppression of the WBC reaction in response to infection after HPEC with mitomycinC and cisplatin is an important detail which should be known to any surgical oncologist in charge of these patients. Myelosuppression is a well-known hematologic side effect of doxorubicin, cisplatin, and mitomycinC [16–18]. Although is well known in the field, that HIPEC is overall well tolerated with acceptable myelosuppression rates compared to the systemic use of chemotherapeutic agents [18, 19], special care should be taken to this attenuated myelosuppressive effect which is not a clinical problem per se but may affect the diagnostic utility of WBC counts. This puts HIPEC treatment in line with other clinical situations, e.g., immunosuppression, old age, transplant patients, where the immune system is not able to react properly, and WBC counts or other serum parameters require critical evaluation.

While myelosuppression can be explained by the systemic effect of locoregional chemotherapy, the underlining mechanism of the secondary inflammation wave and CRP peak remains unclear. However, the clinical consequence is relevant. In the present study, 16% of patients after HIPEC with mitomycinC/doxorubicin underwent a CT scan due to increased CRP levels without diagnosing any postoperative infection. We speculated in a recent study, that prolonged perfusion protocols may trigger a systemic inflammatory response by translocation of intestinal bacterial components [11]. The pathophysiologic mechanism behind this remains, however, still elusive. We observed in this study that patients treated with a 90-min protocol, who also shows depressed WBC and unspecific late CRP elevations, had more organ space infections compared to the short protocol with oxaliplatin. We do interpret this result with the highest care, due to the heterogeneity of groups which could explain this observed difference.

To improve diagnostic accuracy, PCT was introduced earlier for postoperative infection [20]. We share the opinion of these authors that the diagnostic value of serum parameters in the first postoperative days is limited and is highly triggered by the amount and type of surgery. In this critical phase, the experience of the surgeon and particularly the clinical picture of the patient is more relevant, and serum parameters are of limited use to predict complications. However, towards the end of the first postoperative week, when the first peak of surgery-related inflammation flattens, these markers may help to improve patient management. PCT is produced by the C cells of the thyroidal gland and some other cell types upon bacterial infection and is stimulated by bacterial endotoxins and lipopolysaccharides, and indirectly by inflammatory markers, such as tumor necrosis factor-alpha, interleukin-6, and interleukin, and has a high specificity in the diagnosis of bacterial infections and sepsis [21]. In this study, the high specificity of PCT to diagnose infectious complications could be confirmed and was independent from the applied HIPEC protocol. Despite its low sensitivity, the specificity of PCT, which remains unchanged by the perfusion protocol, is an important tool that may be helpful to discriminate between inflammation and infection in the sometimes challenging management of patients after CRS/HIPEC.

We would like to acknowledge the limitations of our study. Overall, the patient cohort includes different primary tumors and therefore the amount of surgery or CRS is not entirely comparable. Some differences in the early postoperative kinetics of the assessed parameters could also be related to this. For example, patients with pseudomyxoma were treated with mitomycinC, which translates into a longer operation time compared to the other protocols. However, the aim of the study, to look at the kinetics of blood and serum parameters, and to assess their diagnostic sensitivity and specificity, in the presence or absence of infection should not be influenced by this heterogeneity. The difference among groups with regard to ICU stay, hospital stay, and infectious complications should not influence the analysis of diagnostic parameters. While we assessed the most commonly used markers, it would be certainly interesting to assess the diagnostic potential of other inflammatory markers such as IL-6, IL-1, or TNF-a to get a deeper insight of the impact of HIPEC on a patient’s physiology.

In conclusion, we analyzed kinetics and the diagnostic value of CRP, WBC, and PCT after uncomplicated and complicated CRS/HIPEC. We identified a major impact on CRP levels and WBC counts, depending on the type of HIPEC protocol. In addition, we propose the use of PCT as a marker for infection which demonstrated to be independent from the treatment and offers a good specificity despite a still low sensitivity. Together our data highlight the complexity of HIPEC treatment which goes beyond technical excellence in the operating room but requires a dedicated holistic care of the surgical oncologist.

## Supplementary Information

**Additional file 1: Figure S1.** The postoperative CRP course is PCI independent. The CRP course for three different PCI groups is plotted in Fig. 1A and illustrates the secondary increase or stable CRP level after CRS/HIPEC. As shown in Suppl. Figure 1B. – D., the HIPEC protocol mainly influences the course of the CRP in all three PCI groups and the main findings remain consistent. Whereas mitomycinC and cisplatin are associated with a CRP increase, after oxaliplatin HIPEC, the CRP decreases almost to normal.

## Data Availability

Human data is stored on a server at the University Hospital of Zurich. All measured serum samples of the patients are stored at −80°C at the University Hospital of Zurich.

## Abbreviations

CRS: Cytoreductive surgery
HIPEC: Hyperthermic intraperitoneal chemotherapy
PCI: Peritoneal cancer index
CC-score: Completeness of cytoreduction score
CRC: Colorectal cancer
CRP: C-reactive protein
PCT: Procalcitonin
WBC: White blood cells
