# Supplementary figures and images for: Serum procalcitonin improves diagnosis of infectious complications after CRS/HIPEC
Source: World J Surg Oncol. 2023 Jan 12;21:5. doi: 10.1186/s12957-022-02884-9 (PMC9835368; doi:10.1186/s12957-022-02884-9)

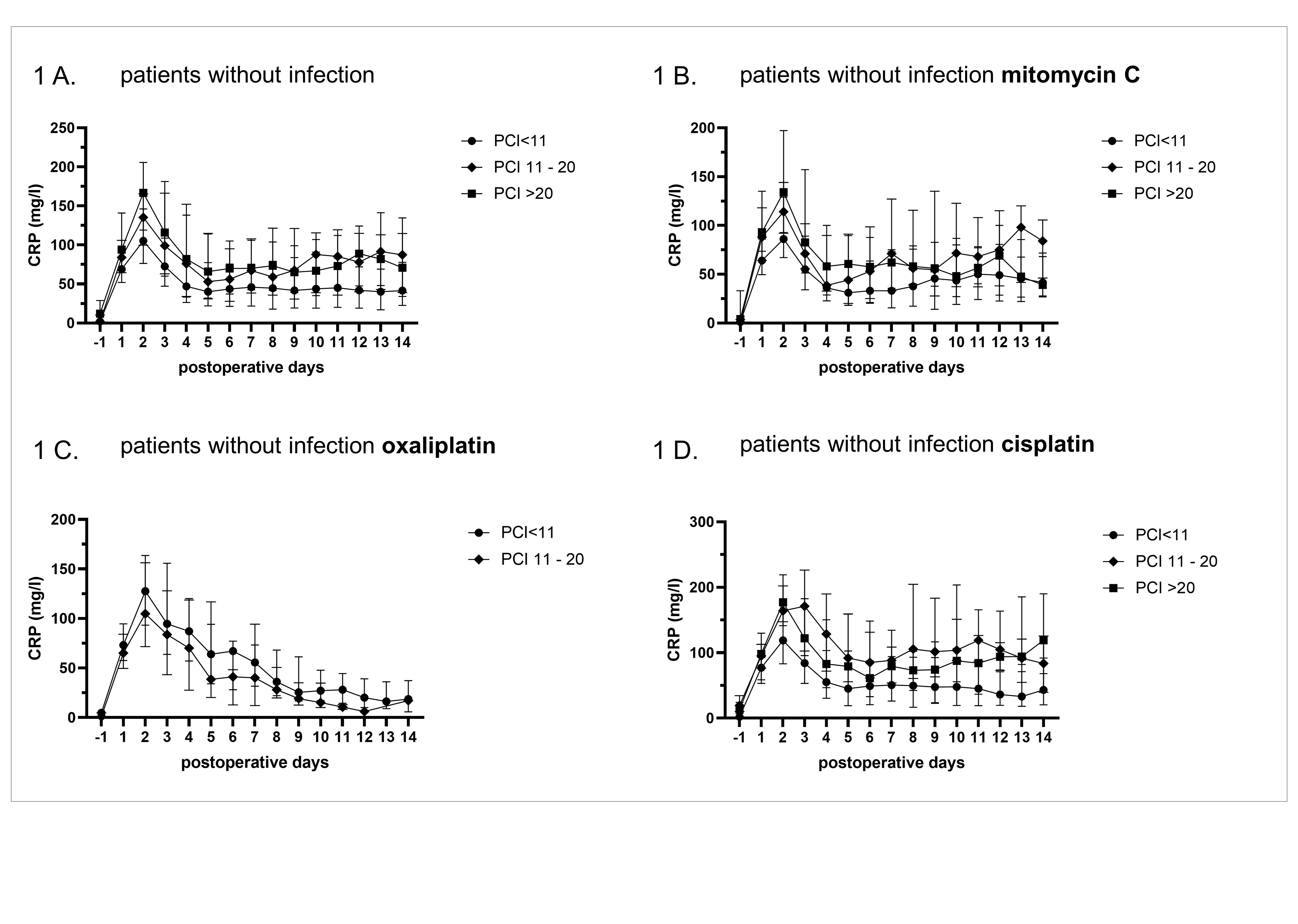

Supplement: Supplementary file 1 — Additional file 1: Figure S1. The postoperative CRP course is PCI independent. The CRP course for three different PCI groups is plotted in Fig. 1A and illustrates the secondary increase or stable CRP level after CRS/HIPEC. As shown in Suppl. Figure 1B. – D., the HIPEC protocol mainly influences the course of the CRP in all three PCI groups and the main findings remain consistent. Whereas mitomycinC and cisplatin are associated with a CRP increase, after oxaliplatin HIPEC, the CRP decreases almost to normal. [file 12957_2022_2884_MOESM1_ESM.jpg]
